# Proximal Humerus Fractures in the Elderly U.S. Population: A Cross-Sectional Study of Treatment Trends and Comparison of Complication Rates after Joint Replacement, Open Reduction and Internal Fixation, and Non-Surgical Management

**DOI:** 10.3390/jcm12103506

**Published:** 2023-05-17

**Authors:** Nike Walter, Dominik Szymski, Moritz Riedl, Steven M. Kurtz, Volker Alt, David W. Lowenberg, Edmund C. Lau, Markus Rupp

**Affiliations:** 1 Department of Trauma Surgery, University Medical Center Regensburg, 93053 Regensburg, Germany; 2 Department of Psychosomatic Medicine, University Medical Center Regensburg, 93053 Regensburg, Germany; 3 Implant Research Center, Drexel University, Philadelphia, PA 19104, USA; 4 Department of Orthopaedic Surgery, Stanford University School of Medicine, Stanford, CA 94063, USA; 5 Exponent Inc., Menlo Park, CA 94025, USA

**Keywords:** complication rates, epidemiology, proximal humerus fractures, risk factors, treatment

## Abstract

Proximal humerus fracture (PHF) treatment remains challenging. Multiple therapy options exist, and the optimal choice of management has been increasingly discussed in the literature. The aim of this study was to (1) analyze trends in the propensity of proximal humerus fracture treatments and (2) compare complication rates after joint replacement, surgical repair, and non-surgical treatment in terms of mechanical complications, union failure, and infection rates. In this cross-sectional study, patients aged 65 years or older with proximal humerus fractures occurring between 1 January 2009 and 31 December 2019 were identified from Medicare physician service claims records. The Kaplan–Meier method with the Fine and Gray adjustment was used to calculate the cumulative incidence rates for malunion/nonunion, infection, and mechanical complications for the following treatment categories: shoulder arthroplasty, open reduction and internal fixation (ORIF), and non-surgical treatment, respectively. Semiparametric Cox regression was performed incorporating 23 demographic, clinical, and socioeconomic covariates to determine risk factors. Between 2009 through 2019, conservative procedures decreased by 0.9%. ORIF procedures decreased from 9.51% (95% CI: 8.7–10.4) to 6.95% (95% CI: 6.2–7.7), whereas shoulder arthroplasties rose from 1.99% (95% CI: 1.6–2.4), to 5.45% (95% CI: 4.8–6.2). PHFs managed through ORIF were associated with higher union failure rates compared to conservatively treated fractures (HR = 1.31, 95% CI: 1.15–1.5, *p* < 0.001). The risk of developing an infection was increased after joint replacement compared to ORIF (2.66% vs. 1.09%, HR = 2.09, 95% CI: 1.46–2.98, *p* < 0.001). Mechanical complications were more common after joint replacement (6.37% vs. 4.85%, HR = 1.66, 95% CI: 1.32–2.09, *p* < 0.001). Complication rates differed significantly across treatment modalities. This should be considered when choosing a management procedure. Vulnerable elderly patient cohorts could be identified, and the optimization of modifiable risk factors might lead to a decrease of complication rates in both surgically and non-surgically treated patients.

## 1. Introduction

Proximal humerus fractures (PHFs) make up 10% of all fractures occurring in patients older than 65 years [1,2]. The prevalence of PHF increases with age. This is especially true in women and represents the third most common osteoporotic fracture type [3,4]. With longer life expectancy in the American population along with the increase in low-energy falls, the incidence of PHFs in the elderly is expected to escalate and potentially pose a high burden on the health care system [5].

Choosing the best treatment for PHF in the elderly is challenging with diverse risks and benefits [2,6]. Management options range from nonoperative treatment to surgical approaches, such as open reduction and internal fixation (ORIF), and arthroplasty alternatives, ranging from hemiarthroplasty and total shoulder arthroplasty to reverse arthroplasty in cases of Neer four-part fractures [7,8]. In fact, probably nowhere else in the body can so many accepted methods of treatment be reported all with fairly similar outcomes. Recently, in the literature, debates have been raised on the optimal treatment modality. A comprehensive meta-analysis incorporated 33 different interventions identified in 31 randomized studies on a total of 1941 patients. Here, the authors concluded that evidence from randomized control trials (RCTs) is insufficient to provide the best choice of treatment for this injury [9]. On the one hand, one randomized trial, which is the largest conducted on PHFs, indicated that non-surgical management should be the standard of care for most patients with a displaced PHF, as surgical treatment did not result in better outcomes [10], whereas in another RCT, it was found that the elderly had more favorable outcomes and cost savings with reverse total shoulder arthroplasty [11]. This highlights the existing controversies regarding the ideal treatment approach of PHFs. To contribute to the evidence of the best management option in terms of reduced complication rates, the aim of this study was two-fold: (1) to analyze the propensity of treatments over the last decade and (2) to compare complication rates after joint replacement, open reduction and internal fixation, and non-surgical procedures in terms of (i) mechanical complications, (ii) union failure, and (iii) fracture-related infection and periprosthetic joint infections, respectively.

## 2. Materials and Methods

For this study, Medicare physician service claims records were used to identify proximal humerus fractures that occurred between 1 January 2009 and 31 December 2019. These records included diagnoses and treatments from various healthcare facilities such as medical offices, outpatient clinics, hospitals, emergency departments, and skilled nursing homes. The Centers for Medicare and Medicaid Services (CMS) compiled these records and made them available for research purposes as the Limited Data Set (LDS) after deidentification. The study was based on physician records associated with a 5% sample of Medicare beneficiaries, equivalent to records from approximately 2.5 million enrollees. To ensure patient privacy, CMS replaced beneficiaries’ identities with synthetic and unique IDs in the LDS data sets, which allowed for the analysis of survivorship and outcomes over time. The study focused on elderly Medicare patients (ages 65 and above) who experienced a proximal humerus fracture during the study period. Patients who were enrolled in a Medicare health maintenance organization (HMO), those younger than 65, and those residing outside of the 50 US states were excluded because the LDS datasets were generated from Medicare fee-for-service enrollees. Since the CMS data were deidentified, it did not require review by the Institutional Review Board.

To identify proximal humerus fractures from physician records, the International Classification of Diseases, Ninth and Tenth Revisions were used. Records submitted before and after 1 October 2015 were recorded in ICD-9-CM and ICD-10-CM, respectively. Only records with a fracture diagnosis listed as the primary diagnosis were retained, and records with the same type of fracture in the previous year were excluded. To ensure the identified fracture was a new fracture, a minimum interval of one year was required from one fracture to the next. For fractures coded using ICD-10, the seventh digit had to be “A” or “B”, indicating a new encounter with that condition. Fractures indicating aftercare for healing of a fracture or malunion/nonunion codes were not counted because they were consistent with pre-existing fractures only. Finally, steps were also taken to check the records in the days after the fracture to ensure that the pattern of service was consistent. For example, we eliminated records which mentioned humerus fracture but had no record of any radiologic imaging of the upper limb or shoulder, no mention of hospital stays, and no mention of any humerus- or shoulder-related procedure. Such “fracture” was likely not a true fracture and was eliminated.

Fracture treatments were categorized into three groups: shoulder arthroplasty, other types of surgeries (ORIF), and non-surgical approaches. To identify these treatments, the study used a set of Common Procedural Terminology (CPT) codes previously established by Bell and co-authors [12]. Patients who did not receive any of these treatments, but were fitted with a sling or other immobilization, were classified as receiving non-operative treatment. The study examined the frequency of these treatments for proximal humerus fractures during the 10-year study period.

Further, several outcome analyses were conducted in this study. Three types of outcomes were investigated. They were: (a) the likelihood of malunion or nonunion following the fracture, (b) the risk of post-fracture infection, and (c) mechanical complication following fracture repairs. Patients were followed from the time of fracture until the outcome was observed, until the end of their enrollment, until death, or until 31 December 2019, whichever occurred first. For malunion or nonunion, patients who received a shoulder arthroplasty after fracture were not tracked since the fractured section was replaced.

Survival analysis techniques were applied to calculate the outcomes. The Kaplan–Meier (KM) method, with the Fine and Gray sub-distribution adaptation to account for competing risk, was performed to calculate the cumulative incidence rate of the malunion/nonunion, infection, and mechanical complications. Post-fracture infection and mechanical complications were analyzed only for patients who received shoulder arthroplasty or other operational treatments such as fixation devices. To investigate outcomes and compare risk among patients receiving different types of treatment, Cox regression was used while also accounting for competing risk. Possible confounding factors were adjusted for in the Cox models, which included demographic, clinical, and several community-level socioeconomic measures as covariates. Demographic factors such as age, sex, race, resident region, and Medicare buy-in were incorporated, while clinical factors consisted of osteoporosis, obesity, diabetes mellitus, rheumatoid disease, chronic kidney disease, tobacco dependence, regular use of anti-coagulant, regular NSAID use, prior osteoporotic fracture, hypertensive disease, ischemic heart disease, cerebrovascular disease, chronic obstructive pulmonary disease (COPD), and congestive heart failure. These conditions were identified from physician records in the one-year period before the fracture, with Table A1 (Appendix A) providing the codes used to identify them.

The socioeconomic measures included population, median household income, percent of population with at least a college education, percent of population in poverty, percent of unemployment, and a measure of the urban–rural character of the county. These measures were obtained from the US Census, the Census Bureau’s American Community Survey (ACS) program, and the Economic Research Service of the USDA (https://www.census.gov/programs-surveys/acs/data.html; https://www.ers.usda.gov/data-products/county-level-data-sets/, accessed on 15 December 2022).

Further, open or closed fractures were characterized, along with whether there was another bone fracture involved in a concomitant fracture incidence. The fracture was also checked to see if it was related to a fall or a vehicle crash.

All data processing and statistical analyses were performed using the SAS statistical software (Version 9.4, Cary, NC, USA) and significance was set at α = 0.05.

## 3. Results

### 3.1. Treatment Trends

**Figure 1:**
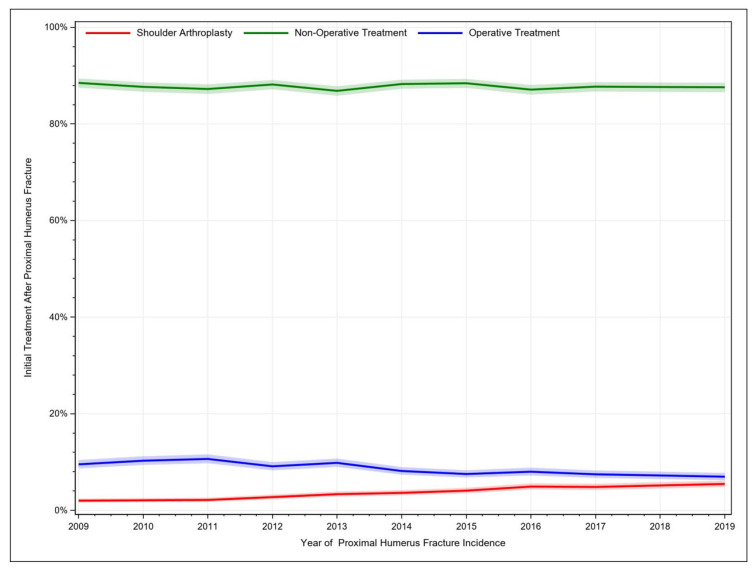
Historic development of treatment procedures for proximal humerus fractures.

For the year 2019, a total of 4329 PHFs was identified from the 5% sample of Medicare beneficiaries. PHFs were mostly managed conservatively. Over the considered time period, non-operative treatment consistently accounted for over 80% of PHF cases but decreased slightly from 88.5% to 87.6%. Additionally, other operative procedures decreased from 9.51% to 6.95%, whereas shoulder arthroplasties after PHF increased from 1.99% to 5.45% (Figure 1, Table 1).

### 3.2. Union Failure

**Figure 2:**
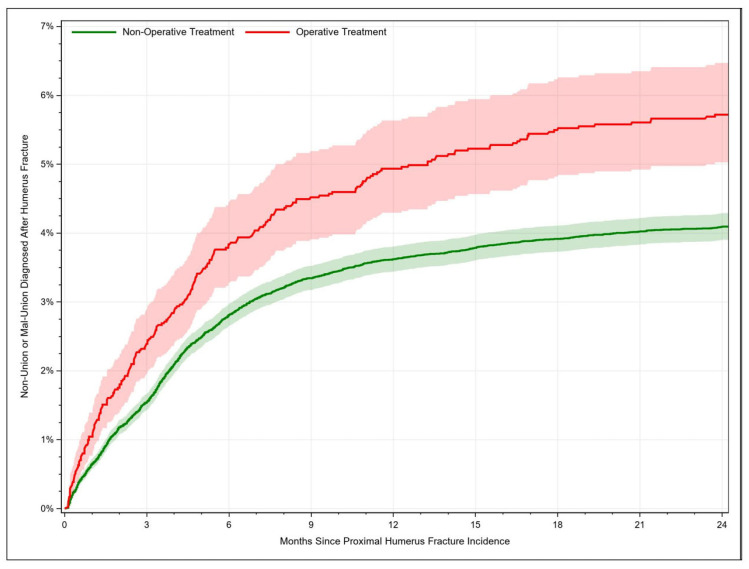
Rate of proximal humerus fracture consolidation failure (malunion or nonunion) depending on time.

The occurrence of union failure after PHF increased with time. For fractures managed conservatively, the union failure rate rose from 0.65% [95% CI: 0.57–0.73] after 1 month to 4.09% [95% CI: 3.90–4.29] after 24 months. Surgically managed PHFs failed to consolidate adequately in 1.05% [95% CI: 0.77–1.39] of the cases after 1 month and in 5.72% [95% CI: 5.03–6.47] of the cases after 24 months (Figure 2). Union failure was significantly less common in patients aged 80+ years (HR = 0.71, 95% CI: 0.62–0.81, χ^2^ = 25.32, *p* < 0.001) compared to patients aged 65–69 years. Additionally, surgically managed fractures were associated with higher union failure rates compared to conservatively treated fractures (HR = 1.31, 95% CI: 1.15–1.5, *p* < 0.001). Other significant risk factors for impaired fracture healing included black race, resident region in the western states, patients with COPD, cerebrovascular disease, congestive heart failure, hypertensive disease, morbid obesity, rheumatoid disease, and those with an open fracture (Table 2).

### 3.3. Fracture-Related Infection and Periprosthetic Joint Infection

**Figure 3:**
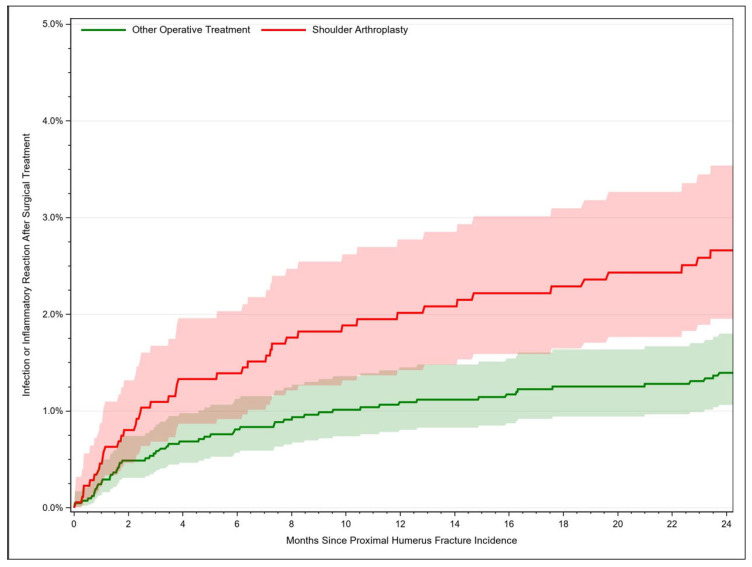
Rate of fracture-related infection after proximal humerus fracture depending on time.

For fractures managed with shoulder arthroplasty, the infection rate rose from 2.02% [95% CI: 1.42–2.77] after 12 months to 2.66% [95% CI: 1.95–3.54] after 24 months. Fracture-related infections occurred in 0.24% [95% CI: 0.13–1.39] of the cases after 1 month, in 1.09% [95% CI: 0.81–1.45] after 12 months and in 1.40% [95% CI: 1.06–1.80] of the cases after 24 months (Figure 3). Thus, the risk of developing an infection after PHF was twice as high after joint replacement compared to osteosyntheses procedures (HR = 2.09, 95% CI: 1.46–2.98, *p* < 0.001). Further risk factors for an infection comprised age older than 75 years compared to 65–69 years, female sex, and sustaining an open fracture (Table 3).

### 3.4. Mechanical Complications

**Figure 4:**
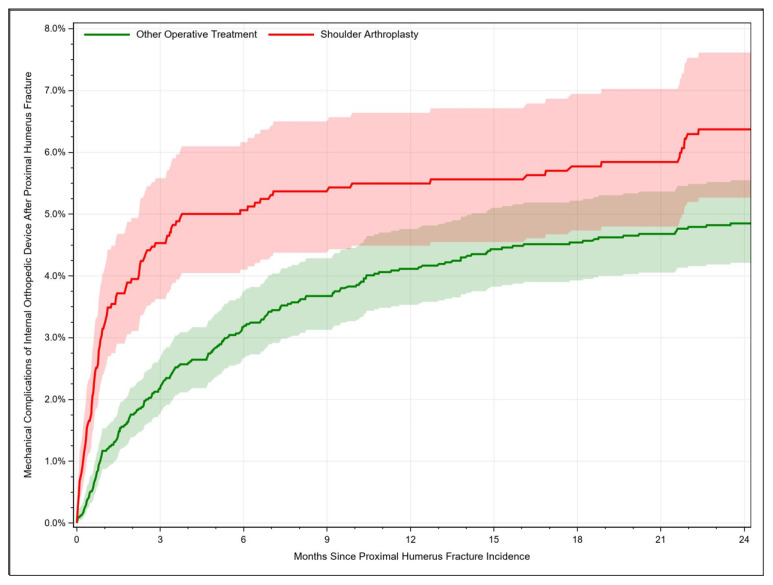
Rate of mechanical complications after treatment of proximal humerus fractures depending on time.

For patients treated with shoulder arthroplasty, mechanical complications occurred in 3.26% [95% CI: 2.50–4.17] of cases after 1 month, whereby the complication rate increased to 6.37% [95% CI: 5.27–7.61] after 24 months. Additionally, mechanical complications after other operative treatment procedures rose from 1.17% [95% CI: 0.87–1.53] after 1 month to 4.85% [95% CI: 4.21–5.55] after 24 months (Figure 4). Hence, the risk was higher when PHFs were managed with joint replacement (HR = 1.66, 95% CI: 1.32–2.09, *p* < 0.001). Neither age nor any of the considered clinical factors were identified as significant risk factors for mechanical complication. Only female sex resulted in a significant lower hazard ratio (HR= 0.67, 95% CI: 0.52–0.87, *p* = 0.003).

## 4. Discussion

In recent years, much attention has been given to the question of the optimal management of proximal humerus fractures. This study provides a comprehensive analysis of trends in treatment procedures and complication rates in terms of (i) mechanical complications, (ii) union failure, and (iii) fracture-related infection or periprosthetic joint infection as well as associated risk-factors.

### 4.1. Increasing Trend of Surgically Managed Proximal Humerus Fractures

An increasing trend in shoulder arthroplasty and a subsequent decrease in other operative treatments were identified. In total, 87.6% of PHF patients were managed conservatively in 2019. In a previous study, PHF patients and the associated treatment were identified in Medicare data from 2005 through 2012. The authors reported an increase in total shoulder arthroplasty from 3% to 17%, and nonoperative treatment was the most common method of treatment with 67% [13]. Additionally, other studies based on a nationwide commercially available administrative claims database (PearlDiver, Inc., Fort Wayne, IN, USA) showed that non-surgical management increased from 80% to 85% during 2010 through 2019 (*p* < 0.001). Further, patients were significantly more likely to undergo a reverse total shoulder arthroplasty (OR = 22.65) compared to total shoulder arthroplasty, ORIF, closed reduction percutaneous pinning, hemiarthroplasty, and intramedullary nailing in 2019 compared to 2010 [14]. However, these trends are not reflected in the literature as more than 70% of publications on PHF deal with surgical treatment modalities (out of which only approximately 3% constitute randomised clinical trials), whereas less than 5% consider non-surgical management [15].

### 4.2. Choice of Treatment

In a comprehensive Cochrane database review pooling data from 31 randomized studies, Handoll and Brorson concluded that there is currently insufficient evidence to determine the most effective treatment method for PHF. The authors found no clinically significant differences in terms of functional outcomes and quality of life when comparing patients treated with surgery and those treated without surgery [9]. Contrary to that, though, we showed that complication rates differed significantly across treatment modalities. Surgically managed fractures were associated with higher union failure rates. The risk of developing an infection after PHF was twice as high after joint replacement compared to osteosynthesis procedures, and mechanical complications were more likely when PHFs were managed with joint replacement. Additionally, other evidence such as higher complication and revision rates has brought into question the surgical treatment of PHF in elderly patients compared to nonoperative management [16]. In patients aged older than 60 years treated with open reduction and internal fixation, complication rates of up to 44% and failure rates of 34% were reported [17]. One systemic review including twelve studies and 514 patients even demonstrated higher rates of malunion (16%) and infection occurrence (4%) compared to our findings [18]. Similarly, Neuhaus and colleagues conducted a study based on data obtained from the National Hospital Discharge Survey (NHDS) determining the risk of an in-hospital adverse event as 4.4 times greater with arthroplasty and 2.7 times greater with open reduction and internal fixation compared to nonoperative treatment in patients aged older than 65 years [19]. However, the results should be interpreted with caution, as the choice of surgical versus non-surgical treatment is likely to be influenced by the underlying type of fracture and thus, especially the higher union failure rate after ORIF may reflect more complex cases.

### 4.3. Limitations

The data used in this study are from the Medicare database, which consists of administrative claims records. As with all administrative claims data, there are limitations for orthopedic outcomes research [20,21]. The study does not have access to the patients’ underlying clinical records, and the accuracy of the analysis depends on the correct coding and management of the data, which cannot be guaranteed. Furthermore, some crucial clinical measures, such as radiologic imaging findings and blood chemistry, are not captured via the diagnosis or procedure codes in these data. Although individual records may contain errors, systematic errors throughout millions of records in the entire Medicare system are unlikely. The broad range of facilities submitting claims and the vast number of records processed by Medicare are unique strengths of the dataset. The information on patient characteristics and complications is assumed to be of high quality due to its relevance for billing and the input of appropriate specialists. The Medicare dataset contains a rich set of relevant parameters that is unmatched by other registry data. However, the analysis could not perform subgroup analyses according to fracture classification (e.g., AO, Neer), and the available CPT codes did not allow for a more detailed discrimination of treatment modalities (e.g., anatomical and reverse total shoulder arthroplasty).

## 5. Conclusions

Complication rates differed significantly across treatment modalities. Surgically managed fractures were associated with higher union failure rates compared to conservatively treated fractures, and the risk of developing an infection after PHF was twice as high after joint replacement compared to osteosyntheses procedures. Mechanical complications were more likely when PHFs were managed with joint replacement. This should be taken into account by surgeons when choosing a management procedure. Vulnerable patient cohorts could be identified, and the optimization of modifiable risk factors in elderly patients might lead to a decrease of complication rates in both surgically and non-surgically treated patients.

## Figures and Tables

**Table 1:** Trends of treatment modalities for proximal humerus fractures.

	Non-Operative Treatment			Operative Treatment			Shoulder Arthroplasty		
Year of Fracture	Treatment Percent	Lower 95% CI	Upper 95% CI	Treatment Percent	Lower 95% CI	Upper 95% CI	Treatment Percent	Lower 95% CI	Upper 95% CI
2009	88.50%	87.51%	89.41%	9.51%	8.66%	10.41%	1.99%	1.61%	2.44%
2010	87.67%	86.66%	88.61%	10.26%	9.39%	11.19%	2.06%	1.67%	2.52%
2011	87.23%	86.20%	88.19%	10.63%	9.73%	11.57%	2.14%	1.73%	2.60%
2012	88.17%	87.17%	89.09%	9.10%	8.27%	9.98%	2.73%	2.27%	3.25%
2013	86.85%	85.83%	87.80%	9.82%	8.97%	10.71%	3.33%	2.84%	3.89%
2014	88.26%	87.30%	89.16%	8.13%	7.37%	8.95%	3.61%	3.09%	4.17%
2015	88.43%	87.46%	89.32%	7.51%	6.77%	8.30%	4.06%	3.52%	4.67%
2016	87.11%	86.07%	88.07%	7.98%	7.20%	8.82%	4.91%	4.29%	5.58%
2017	87.72%	86.71%	88.66%	7.45%	6.70%	8.25%	4.83%	4.22%	5.49%
2018	87.64%	86.63%	88.59%	7.21%	6.46%	8.00%	5.15%	4.52%	5.83%
2019	87.60%	86.58%	88.54%	6.95%	6.22%	7.73%	5.45%	4.80%	6.15%

**Table 2:** Risk factors for union failure of proximal humerus fractures. HR = hazard ratio. * *p* < 0.05.

Factor	Wald ChiSq	*p*-Value	HR	Lower HR	Upper HR	Chi-Square	*p*-Value
Race Black vs. White Other vs. White	14.17	<0.001	0.56 0.83	0.41 0.65	0.77 1.06	12.58 2.26	<0.001 * 0.133
Resident Region Midwest vs. South	16.69	<0.001	1.12	0.99	1.26	3.40	0.065
Northeast vs. South			0.87	0.76	1.00	3.76	0.053
West vs. South			1.17	1.01	1.36	4.43	0.035 *
Female sex	0.61	0.436	0.96	0.86	1.07	0.61	0.436
Operative vs. Non-Operative treatment	16.14	<0.001	1.31	1.15	1.50	16.14	<0.001 *
Anticoagulant Use	1.22	0.269	0.89	0.73	1.09	1.22	0.269
COPD	4.71	0.030	1.15	1.01	1.30	4.71	0.030 *
Cerebrovascular Disease	5.85	0.016	0.84	0.72	0.97	5.85	0.016 *
Chronic Kidney Disease	1.23	0.267	1.08	0.94	1.25	1.23	0.267
Concomitant Fracture	1.03	0.309	1.07	0.94	1.23	1.03	0.309
Congestive Heart Failure	8.43	0.004	0.80	0.69	0.93	8.43	0.004 *
Diabetes Mellitus	3.82	0.051	1.11	1.00	1.23	3.82	0.051
Fall Related Fracture	0.09	0.765	0.98	0.88	1.10	0.09	0.765
Hypertensive Disease	15.43	<0.001	1.23	1.11	1.36	15.43	<0.001 *
Insulin Use	1.56	0.211	1.24	0.89	1.74	1.56	0.211
Ischemic Heart Disease	0.82	0.364	1.06	0.94	1.19	0.82	0.364
Morbid Obesity	6.99	0.008	1.51	1.11	2.06	6.99	0.008 *
Open Fracture	4.72	0.030	1.66	1.05	2.61	4.72	0.030 *
Osteoporotic Fracture	0.94	0.333	1.16	0.86	1.58	0.94	0.333
Osteoporosis	3.66	0.056	1.15	1.00	1.33	3.66	0.056
Rheumatoid Disease	9.17	0.002	1.43	1.13	1.80	9.17	0.002 *
Tobacco Dependence	0.95	0.330	1.16	0.86	1.56	0.95	0.330
Year of Fracture	1.21	0.271	1.01	0.99	1.02	1.21	0.271
% College Degree	0.00	0.950	1.00	0.99	1.01	0.00	0.950
% Poverty	0.00	0.996	1.00	0.98	1.02	0.00	0.996
County Population	0.28	0.596	1.00	1.00	1.00	0.28	0.596
Median HH Income	0.01	0.929	1.00	0.97	1.03	0.01	0.929

**Table 3:** Risk factors for an infection after treatment of proximal humerus fractures. HR = hazard ratio. * *p* < 0.05.

Factor	Wald ChiSq	*p*-Value	HR	Lower HR	Upper HR	Chi-Square	*p*-Value
Age at Fracture 70–74 vs. 65–69	19.33	<0.001	1.00	0.61	1.62	0.00	0.987
75–79 vs. 65–69			0.57	0.32	0.99	3.95	0.047 *
80+ vs. 65–69			0.36	0.21	0.63	12.72	<0.001 *
Race Black vs. White	0.77	0.679	0.92	0.21	3.93	0.01	0.905
Other vs. White			0.53	0.13	2.20	0.76	0.383
Resident Region Midwest vs. South	3.18	0.364	0.94	0.57	1.57	0.05	0.818
Northeast vs. South			1.12	0.60	2.08	0.13	0.719
West vs. South			1.53	0.91	2.55	2.58	0.108
Female sex	5.36	0.021	0.61	0.40	0.93	5.36	0.021 *
Shoulder Arthroplasty vs. Other Operative Treatment	16.38	<0.001	2.09	1.46	2.98	16.38	<0.001 *
Anticoagulant Use	0.40	0.526	1.30	0.57	2.97	0.40	0.526
COPD	0.45	0.503	1.21	0.70	2.08	0.45	0.503
Cerebrovascular Disease	0.18	0.673	1.15	0.61	2.17	0.18	0.673
Chronic Kidney Disease	2.95	0.086	1.67	0.93	2.98	2.95	0.086
Concomitant Fracture	0.02	0.880	0.96	0.55	1.68	0.02	0.880
Congestive Heart Failure	0.02	0.898	1.05	0.53	2.08	0.02	0.898
Diabetes Mellitus	1.34	0.247	0.77	0.49	1.20	1.34	0.247
Fall Related Fracture	0.27	0.606	0.88	0.55	1.42	0.27	0.606
Hypertensive Disease	0.01	0.926	0.98	0.65	1.48	0.01	0.926
Ischemic Heart Disease	0.00	0.991	1.00	0.59	1.69	0.00	0.991
Morbid Obesity	0.94	0.333	1.73	0.57	5.22	0.94	0.333
Open Fracture	2059.74	<0.001	0.00	0.00	0.00	2059.74	<0.001 *
Osteoporotic Fracture	0.45	0.501	0.51	0.07	3.56	0.45	0.501
Osteoprosis	1.85	0.174	0.56	0.25	1.29	1.85	0.174
Rheumatoid Disease	0.03	0.853	0.90	0.28	2.86	0.03	0.853
Tobacco Dependence	0.00	0.996	1.00	0.31	3.25	0.00	0.996
Year of Fracture	0.97	0.325	0.97	0.92	1.03	0.97	0.325
% College Degree	0.12	0.724	1.01	0.96	1.05	0.12	0.724
% Poverty	2.25	0.134	0.93	0.85	1.02	2.25	0.134
County Population	0.01	0.911	1.00	0.99	1.01	0.01	0.911
Median HH Income	0.15	0.696	0.98	0.87	1.10	0.15	0.696

## Data Availability

The datasets used and/or analyzed during the current study are available from the corresponding author on reasonable request.

## Appendix A

**Table A1:** Clinical indicator used.

Condition	ICD-9	ICD-10
Osteoporosis	733.0x	M80.x, M81.x
Osteoporotic Fracture	733.1x	M80.x, M8.4x
Diabetes mellitus	250.x	E08.x, E09.x, E10.x, E11.x, E13.x
Morbid Obesity	278.01, V85.4x	E66.01x, E66.2x, Z68.4x
Rheumatoid Disease	714.0x	M05.4x, M05.5x, M05.7x, M05.8x, M05.9x, M06.0x, M06.2x, M06.3x, M06.8x, M06.9x
Chronic Kidney Disease	585.x	N18.x
Tobacco Dependency	305.1x	F17.20x, F17.21x, F17.22x, F17.29x
Anticoagulant Use	V58.61x	Z79.01x
Opioid Use	304.0x, 304.7x, 305.5x	F11.1x, F11.2x, F11.9x
NSAID Use	V586.4x	Z79.1x
Hypertension	401.x, 402.x, 403.x, 404.x, 405.x	I11.x, I12.x, I13.x, I14.x, I15.x
Ischemic Heart Disease	410.x, 411.x, 412.x, 413,x, 414.x	I21.x, I22.x, I23.x, I24.x, I25.x
Cerebrovascular Disease	430.x, 431.x, 432.x, 433.x, 434.x, 435.x, 436.x, 437.x, 438.x	I60.x, I61.x, I62.x, I63.x, I64.x, I65.x, I66.x, I67x, I68.x, I69.x
COPD	491.2x, 493.2x, 496.x, 518.8x	J44.x
Congestive Heart Disease	428.0x, 428.1x, 428.2x, 428.3x, 428.4x, 428.9x	I50.x
Fall Related	E88.x, E98.7x	W0x, W1x
Vehicle Crash Related	E81x, E820x, E821x, E822x, E823x, E824x, E825x	V0x, V1x, V2x, V3x, V4x, V5x, V6x, V7x

## References

[1] Kim S.H., Szabo R.M., Marder R.A. (2012). Epidemiology of humerus fractures in the United States: Nationwide emergency department sample, 2008. Arthritis Care Res..

[2] Sabharwal S., Patel N.K., Griffiths D., Athanasiou T., Gupte C.M., Reilly P. (2016). Trials based on specific fracture configuration and surgical procedures likely to be more relevant for decision making in the management of fractures of the proximal humerus: Findings of a meta-analysis. Bone Jt. Res..

[3] Johnell O., Kanis J. (2005). Epidemiology of osteoporotic fractures. Osteoporos. Int..

[4] Launonen A.P., Lepola V., Saranko A., Flinkkilä T., Laitinen M., Mattila V.M. (2015). Epidemiology of proximal humerus fractures. Arch. Osteoporos..

[5] Court-Brown C.M., Caesar B. (2006). Epidemiology of adult fractures: A review. Injury.

[6] Floyd S.B., Thigpen C., Kissenberth M., Brooks J.M. (2020). Association of Surgical Treatment with Adverse Events and Mortality Among Medicare Beneficiaries with Proximal Humerus Fracture. JAMA Netw. Open.

[7] Iacobellis C., Berizzi A., Biz C., Camporese A. (2015). Treatment of proximal humeral fractures with reverse shoulder arthroplasty in elderly patients. Musculoskelet. Surg..

[8] Baker H.P., Gutbrod J., Strelzow J.A., Maassen N.H., Shi L. (2022). Management of Proximal Humerus Fractures in Adults-A Scoping Review. J. Clin. Med..

[9] Handoll H.H.G., Brorson S. (2015). Interventions for treating proximal humeral fractures in adults. Cochrane Database Syst. Rev..

[10] Handoll H., Brealey S., Rangan A., Keding A., Corbacho B., Jefferson L., Chuang L.-H., Goodchild L., Hewitt C., Torgerson D. (2015). The ProFHER (PROximal Fracture of the Humerus: Evaluation by Randomisation) trial—A pragmatic multicentre randomised controlled trial evaluating the clinical effectiveness and cost-effectiveness of surgical compared with non-surgical treatment for proximal fracture of the humerus in adults. Health Technol. Assess..

[11] Chalmers P.N., Slikker W., Mall N.A., Gupta A.K., Rahman Z., Enriquez D., Nicholson G.P. (2014). Reverse total shoulder arthroplasty for acute proximal humeral fracture: Comparison to open reduction-internal fixation and hemiarthroplasty. J. Shoulder Elb. Surg..

[12] Bell J.-E., Leung B.C., Spratt K.F., Koval K.J., Weinstein J.D., Goodman D.C., Tosteson A.N.A. (2011). Trends and variation in incidence, surgical treatment, and repeat surgery of proximal humeral fractures in the elderly. J. Bone Jt. Surg. Am..

[13] Han R.J., Sing D.C., Feeley B.T., Ma C.B., Zhang A.L. (2016). Proximal humerus fragility fractures: Recent trends in nonoperative and operative treatment in the Medicare population. J. Shoulder Elb. Surg..

[14] Patel A.H., Wilder J.H., Ofa S.A., Lee O.C., Savoie F.H., O’Brien M.J., Sherman W.F. (2022). Trending a decade of proximal humerus fracture management in older adults. JSES Int..

[15] Slobogean G.P., Johal H., Lefaivre K.A., MacIntyre N.J., Sprague S., Scott T., Guy P., Cripton P.A., McKee M., Bhandari M. (2015). A scoping review of the proximal humerus fracture literature. BMC Musculoskelet. Disord..

[16] Wu E.J., Zhang S.E., Truntzer J.N., Gardner M.J., Kamal R.N. (2020). Cost-Minimization Analysis and Treatment Trends of Surgical and Nonsurgical Treatment of Proximal Humerus Fractures. J. Hand Surg. Am..

[17] Barlow J.D., Logli A.L., Steinmann S.P., Sems S.A., Cross W.W., Yuan B.J., Torchia M.E., Sanchez-Sotelo J. (2020). Locking plate fixation of proximal humerus fractures in patients older than 60 years continues to be associated with a high complication rate. J. Shoulder Elb. Surg..

[18] Sproul R.C., Iyengar J.J., Devcic Z., Feeley B.T. (2011). A systematic review of locking plate fixation of proximal humerus fractures. Injury.

[19] Neuhaus V., Bot A.G.J., Swellengrebel C.H.J., Jain N.B., Warner J.J.P., Ring D.C. (2014). Treatment choice affects inpatient adverse events and mortality in older aged inpatients with an isolated fracture of the proximal humerus. J. Shoulder Elb. Surg..

[20] Pugely A.J., Martin C.T., Harwood J., Ong K.L., Bozic K.J., Callaghan J.J. (2015). Database and Registry Research in Orthopaedic Surgery: Part 2: Clinical Registry Data. J. Bone Jt. Surg. Am..

[21] Pugely A.J., Martin C.T., Harwood J., Ong K.L., Bozic K.J., Callaghan J.J. (2015). Database and Registry Research in Orthopaedic Surgery: Part I: Claims-Based Data. J. Bone Jt. Surg. Am..

